# A Small-Molecule Inhibitor of RAD51 Reduces Homologous Recombination and Sensitizes Multiple Myeloma Cells to Doxorubicin

**DOI:** 10.3389/fonc.2014.00289

**Published:** 2014-10-30

**Authors:** David A. Alagpulinsa, Srinivas Ayyadevara, Robert Joseph Shmookler Reis

**Affiliations:** ^1^McClellan Veterans Medical Center, Central Arkansas Veterans Healthcare System, Little Rock, AR, USA; ^2^Department of Geriatrics, University of Arkansas for Medical Science, Little Rock, AR, USA

**Keywords:** multiple myeloma, RAD51, H2AX, recombinase, homologous recombination, chemoresistance, doxorubicin, B02

## Abstract

We previously reported high expression of RAD51 and increased homologous recombination (HR) rates in multiple myeloma (MM) cells, and showed that genomic instability and disease progression are commensurate with HR levels. Moreover, high RAD51 expression *in vivo* is associated with chemoresistance and poor patient survival. Doxorubicin (DOX) is one of the most widely used drug treatments in MM chemotherapy. DOX is cytotoxic because it induces DNA double-strand breaks, which can be repaired by RAD51-mediated HR; activation of this pathway thus contributes to resistance. To investigate the role of RAD51 in MM drug resistance, we assessed the ability of B02, a small-molecule inhibitor of RAD51, to enhance DOX sensitivity of MM cells. Combining low-toxicity doses of DOX and B02 resulted in significant synthetic lethality, observed as increased apoptosis and reduced viability compared to either agent alone, or to the product of their individual effects. In contrast, the combination did not produce significant synergy against normal human CD19^+^ B cells from peripheral blood. DOX induced RAD51 at both mRNA and protein levels, while arresting cells in S and G2. DOX treatment also increased the number of RAD51 foci, a marker of HR repair, so that the fraction of cells with ≥5 foci rose fourfold, whereas γH2AX foci rose far less, implying that most new breaks are repaired. When B02 treatment preceded DOX exposure, the induction of RAD51 foci was severely blunted, whereas, γH2AX foci rose significantly relative to basal levels or either agent alone. In MM cells carrying a chromosomally integrated reporter of HR repair, DOX increased HR events while B02 inhibition of RAD51 blocked the HR response. These studies demonstrate the crucial role of RAD51 in protecting MM cells from genotoxic agents such as DOX, and suggest that specific inhibition of RAD51 may be an effective means to block DNA repair in MM cells and thus to enhance the efficacy of chemotherapy.

## Introduction

Multiple myeloma (MM) is a plasma cell cancer arising from malignant transformation of post-follicular B cells. The disease is the second most common hematologic malignancy, accounting for about 15% of all new cases of such cancers. It is essentially incurable in the majority of patients, accounting for about 20% of all deaths from hematologic malignancies (1). A major hallmark of MM cells is their extensive genomic instability, accompanied by molecular heterogeneity at many levels (2, 3). Although the mechanisms underlying these abnormalities are not well understood, aberrant DNA repair mechanisms have been implicated. We previously showed high-level expression of the RAD51 recombinase and its paralogs in MM cell lines *in vitro*, and also in primary bone-marrow aspirates from MM patients. We demonstrated that *Rad51* gene induction in MM cell lines increases homologous recombination (HR) activity and mediates genomic instability and disease progression, including development of chemotolerance (4). HR is an essential cellular process, enabling cells to cope with genotoxic stress by repairing DNA interstrand cross-links (ICLs), stalled/damaged replication forks, and double-strand breaks (DSBs) with relatively high fidelity (5, 6). RAD51 polymerizes onto single-strand overhangs at resected DNA breaks to form a nucleofilament, which initiates invasion of homologous duplexes leading to reciprocal and non-reciprocal DNA strand exchanges (7). It appears to be the pivotal protein driving the HR process, since its overexpression elicits aberrant recombination events (8, 9) while its suppression lowers recombination frequency (4). A growing body of evidence suggests that high expression of RAD51 correlates with an enhanced propensity of tumor cells for invasiveness (10), aggressiveness (11), poor prognosis (12–17), and resistance to DNA damage induced by chemotherapeutic drugs (17–21) or radiotherapy (22). Recently, high RAD51 expression was reported to have a negative prognostic value for both event-free and overall survival of MM patients (23). Targeting RAD51 has thus been proposed as a potential anti-cancer treatment, and downregulation of RAD51 by siRNA has been shown to selectively increase the chemotherapeutic sensitivity of human cancer cells relative to normal cells (24).

Doxorubicin is one of the most widely used drugs in chemotherapy regimens for MM. Doxorubicin (DOX) intercalates between stacked DNA base pairs, inhibiting topoisomerase II, and subsequently inducing DNA DSBs (25) preferentially in replicating cells (26). HR and nucleotide excision repair pathways (which are primarily active in replicating cells) are thus critical for the repair of these lesions (27). Consequently, constitutive upregulation of RAD51 and HR in cancer cells has the potential to create resistance to DOX or other genotoxic drugs. Non-homologous end-joining (NHEJ), the other major pathway for DSB-repair, appears to be disrupted in MM cells. As a result, MM may be particularly dependent on HR, as has been observed for repair of radiation-induced DSBs when NHEJ is inhibited (28). MM-cell reliance on RAD51-dependent HR repair, to survive genotoxic and/or replicative stresses, could be clinically exploited for synthetic lethality or to widen the therapeutic-dose window, by combining DNA damaging agents such as DOX with inhibitors of HR repair. There are precedents in which agents that indirectly target the function and/or expression of RAD51 were found to improve the efficacy of MM radio- and chemotherapy (29, 30). However, no studies have specifically examined the role played by RAD51 in MM chemoresistance, particularly to DOX, or the therapeutic potential of RAD51 small-molecule inhibitors in this disease.

Huang and co-workers identified B02 as a specific inhibitor of human RAD51 recombinase (31) and demonstrated that B02 blocks HR repair in human embryonic kidney (HEK) and breast cancer cells and increases their sensitivity to a wide range of DNA damaging agents (32, 33). Also, Maes et al. reported that B02 enhances DNA damage and apoptosis induced by decitabine in MM cells (34). Here, we investigated the involvement of RAD51-mediated HR repair in MM-cell response to DOX, asking whether B02 will sensitize MM cells to this treatment. We show that DOX elicits dose-dependent induction of RAD51 expression at both mRNA and protein levels, and that treated MM cells arrest in the S and G2 cell-cycle phases wherein HR predominantly occurs. Treatment with DOX alone evokes a marked increase in nuclear RAD51 focus formation, an indicator of RAD51-mediated repair, while the level of unrepaired DNA damage (indicated by γH2AX foci) remains relatively constant. Pre-treatment with B02, however, upsets that balance, blocking formation of DOX-induced RAD51 foci and elevating measures of DNA damage. Consequently, combined treatment with B02 and DOX results in greater-than-additive cytotoxicity to MM cells. This study demonstrates that RAD51 is essential for “normal levels” of MM-cell resistance to DOX treatment, so that direct inhibition of RAD51 could be an effective addition to clinical regimens, enhancing the efficacy of genotoxic chemotherapies.

## Materials and Methods

### Cell culture and reagents

The human MM cell lines NCI-H929 (H929), RPMI 8226, ARP-1, and U266 (provided by Dr. Shmuel Yaccoby, Myeloma Institute for Research and Therapy, University of Arkansas for Medical Sciences) and MM.1S (obtained from the ATCC) were maintained in RPMI-1640 medium with L-glutamine and NaHCO_3_ (ATCC) containing 10% FBS (ATCC), 100 U/mL of penicillin, and 100 μg/mL of streptomycin (Sigma-Aldrich). All cultures were maintained at 37°C, 5% CO_2_, and 70% relative humidity. MM.1S-DR.GFP cells (from N. Bahlis, University of Calgary, Canada) were maintained in the same medium, supplemented with 2 μg/mL puromycin (Sigma-Aldrich). DOX-HCl and B02 (Sigma-Aldrich) were dissolved in DMSO and diluted in cell culture medium for cell treatment. Normal human peripheral blood CD19^+^ B cells (ZenBio) were cultured in ZenBio Lymphocyte Medium.

### Cell proliferation assay

Proliferation of MM-cell lines was monitored by the WST-1 (Clontech) colorimetric cell-count assay. MM cell lines were seeded in 96-well plates at ~8000 cells/well. The cells were treated with or without B02 (10 μM) for 1 h, followed by treatment with vehicle (DMSO) or DOX (20–160 nM) for 72 h. WST-1 reagent was added to the culture medium in each well at a 1:10 ratio, and incubation continued at 37°C for 4 h. Relative cell number was estimated from absorbance at 450 nm using a spectrophotometer (Molecular Devices Corp., CA, USA), and the percentage viability of cells calculated relative to vehicle treatment (set as 100% viability for dose-response curves).

To determine the effect of *Rad51* siRNA on MM-cell chemosensitivity, MM.1S and H929 cells were transfected with an anti-*Rad51*-specific siRNA construct, or a scrambled-sequence control, as previously described (4), using the Amaxa^®^ Nucleofector^®^ II (Lonza, Germany) and Nucleofector Kit V (Amaxa VCA-1003) according to the manufacturer’s protocol. At 24 h after transfection, cells were counted and seeded in 96-well plates (~8000 cells/well) and treated as indicated. Cell viability was then assayed after 72 h by WST-1 as described above.

For viability assays on normal human peripheral blood B cells, CD19^+^ B cells were placed in 96-well plates in lymphocyte medium (~8000 cells/well) and treated with DOX ± B02 as described above. Cell count was estimated by WST-8 assay (Sigma CCK-8) at 72 h according to the manufacturer’s protocol, and viability expressed relative to vehicle treatment (set as 100% viability).

### Colony formation assay

Cells were treated with B02 (10 μM) for 1 h, followed by addition of vehicle (DMSO) or DOX (80 nM). After 24 h, cells were washed in fresh culture medium and counted. Cells were then suspended at ~1000 cells/mL in 0.5% Sea-Plaque agarose (Lonza) in RPMI-1640 medium (maintained as liquid at 41°C), and overlaid onto a bed of solidified 0.6% agarose in RPMI-1640 medium in six-well plates. Culture medium (~1 ml per well) was added to keep cells submerged. Plates were incubated for 14–21 days until colonies were visible on viewing through an inverted microscope, and colonies of ≥50 cells were counted. The number of colonies, divided by that observed for vehicle alone, is the fraction surviving treatment.

### Analyses of cell-cycle phases, phosphorylated histone H3, and apoptosis by flow cytometry

For cell-cycle distribution, 70% ethanol-fixed cells were stained with propidium iodide (PI) containing RNase A (FxCycle PI/RNase, Invitrogen) and analyzed for DNA content using FACS-Diva Software (BD Biosciences). For detection of phosphorylated histone H3 (ser10; pHH3) and DNA content, ethanol-fixed cells were treated with 0.25% Triton X-100, washed, and incubated with Alexa Fluor 488-conjugated anti-pHH3 (BioLegend, clone 11D8) in BioLegend Cell Staining Buffer (5 μL per 1 × 10^6^ cells), and stained with PI/RNase A. For apoptosis assays, Annexin V-FITC/PI apoptosis detection kit (Affymetrix, eBioscience) was used.

### Quantitative RT-PCR

Levels of RAD51 mRNA were determined by quantitative real-time polymerase chain reaction (qRT-PCR). Total RNA was extracted from cells after each indicated treatment using Qiagen RNAeasy Mini Kit (Qiagen) and cDNA reverse-transcribed from RNA using SuperScript First Strand cDNA synthesis kit (Invitrogen). Amplified PCR products were detected using a SYBR Green PCR Master Mix (Roche). Threshold cycle number (T_c_) for each sample was normalized to T_c_ for cytoplasmic β-actin. Primers used for amplification were *Rad51* forward (F) and reverse (R) primers: 5′-CAATGCAGATGCAGCTTGAA-3′ and 5′-CCTTGGCTTCACTAATTCCCT-3′, respectively; and β*-actin* F and R primers: 5′-CATCTTGGCCTCACTGTCCA-3′ and 5′-GGGCCGGACTCATCGTATT-3′, respectively.

### Western blotting

After the indicated treatments, cells were lysed at 0°C by mild sonication (3 × 10 s) in RIPA buffer (Santa Cruz Biotech) plus protease inhibitors (Sigma). Protein concentrations were determined by BCA assay (Thermo Scientific) and equivalent protein amounts subjected to SDS-PAGE, transferred to polyvinyl difluoride (PVDF) membranes and probed with mouse monoclonal antibody to RAD51 (Millipore). Membranes were washed in TBST, incubated with HRP-conjugated goat anti-mouse IgG (Santa Cruz Biotechnology), washed again, and signal detected by chemiluminescence using ECL detection reagent (Bio-Rad). Antibodies were removed from the membrane by incubation in a solution containing 2% (w/v) sodium docecylsulfate, 62.5 mM Tris-HCl pH6.8, and 0.7% (w/w) β-mercaptoethanol for 15 min at 50°C and the membrane re-probed with primary antibody to actin (Santa Cruz Biotechnology) and signal detected as described above. Band intensities were quantified using Quantity One software (Bio-Rad) and RAD51 intensity in each lane was expressed relative to the corresponding actin band and normalized to vehicle treatment.

### Immunofluorescence of cells

Cells were treated with or without B02 (20 μM) for 6 h followed by treatment with vehicle (DMSO) or DOX (160 nM) for a further 6 h. Cells were then washed free of external drugs and incubated in fresh RPMI-1640 medium for a further 24 h. Cells were harvested, centrifuged onto glass slides (Cytospin 4, Thermo Scientific), and fixed in 4% paraformaldehyde in PBS, pH 7.4, for 12 min at 22°C, followed by three 5-min washes in PBS. Cells were permeabilized (0.1% Triton X-100 in PBS, 15-min), washed in PBS (3×, 5 min each), and incubated 1 h at 22°C with blocking buffer (1.5% BSA in PBS). They were reacted >12 h at 4°C with primary antibodies [1:1000 goat polyclonal anti-RAD51 IgG (Santa Cruz Biotech.); or 1:1000 mouse monoclonal anti-γH2AX/ser139, clone JBW301 (Millipore)], washed in PBS (3×, 5 min each), and incubated 1 h at 22°C in the dark, with appropriate secondary antibodies [bovine anti-goat IgG Alexa Fluor 488 for RAD51, or goat anti-mouse IgG Alexa Fluor 594 for γH2AX (Jackson ImmunoResearch)]. Cells were washed 3× in PBS and mounted under coverslips with Prolong Antifade plus DAPI. Images were acquired with an LSM 510 Zeiss confocal laser-scanning microscope with a 63× oil objective. For quantitative analysis, ≥100 cells from each group were chosen at random and nuclei counted manually to determine the percentage positive for RAD51 and/or γH2AX (i.e., having ≥5 discrete foci/nucleus). Results were averaged from ≥3 biological replicates. Intensities of foci (integrated densities per nucleus) were measured using ImageJ software, with subtraction of background peripheral to each nucleus.

### HR assay

MM.1S cells containing a chromosomally integrated HR substrate, a “DR-GFP” reporter (from N. Bahlis, University of Calgary, Canada) were used for HR assay (29, 35). Transient infection with an adenovirus expressing I-*Sce*I endonuclease, AdNGUS24i (from N. Bahlis, University of Calgary, and A. Nepveu, McGill University, Canada), generates a DSB at an I-*Sce*I site within a mutant GFP (Sce-GFP) copy in tandem with a 5′- and 3′-truncated GFP gene (iGFP) (36). HR repair of the DSB, templated by the iGFP partial-repeat copy, restores a functional GFP gene whose expression is detectable by flow cytometry. MM.1S cells stably expressing the integrated DR-GFP were cultured 24 h in medium ± AdNGUS24i viral particles, rinsed, and resuspended in fresh culture medium ± B02 (10 μM) or DOX (160 nM) and incubated 24 h. Percent live cells (PI^−^, i.e., impermeable to PI), and the fraction of PI^−^ cells that were also GFP^+^, were measured by flow cytometry to estimate the frequency of HR repair.

### Statistical analysis

All experiments were carried out with at least three samples per group, and repeated at least twice. Data are expressed as mean ± SEM. GraphPad Prism software (Prism ver. 6, San Diego, CA, USA) and Excel were used for statistical analysis. Statistical significances observed between groups were calculated by two-tailed *t*-tests, or by ANOVA with Tukey’s *post hoc* test for comparisons of more than two groups. Two-tailed heteroscedastic *t*-tests were used to determine significance of differences in DOX effects on RAD51 protein levels, since sample size was insufficient to ensure adherence to a Gaussian distribution, or to estimate variance with high confidence. Uncorrected *p* values are presented, to permit reader discretion in defining a threshold between full Bonferroni correction and the *p* < 0.05 level denoting nominal statistical significance.

## Results

### Disruption OF RAD51 potentiates the sensitivity of MM cell lines to doxorubicin

**Figure 1:**
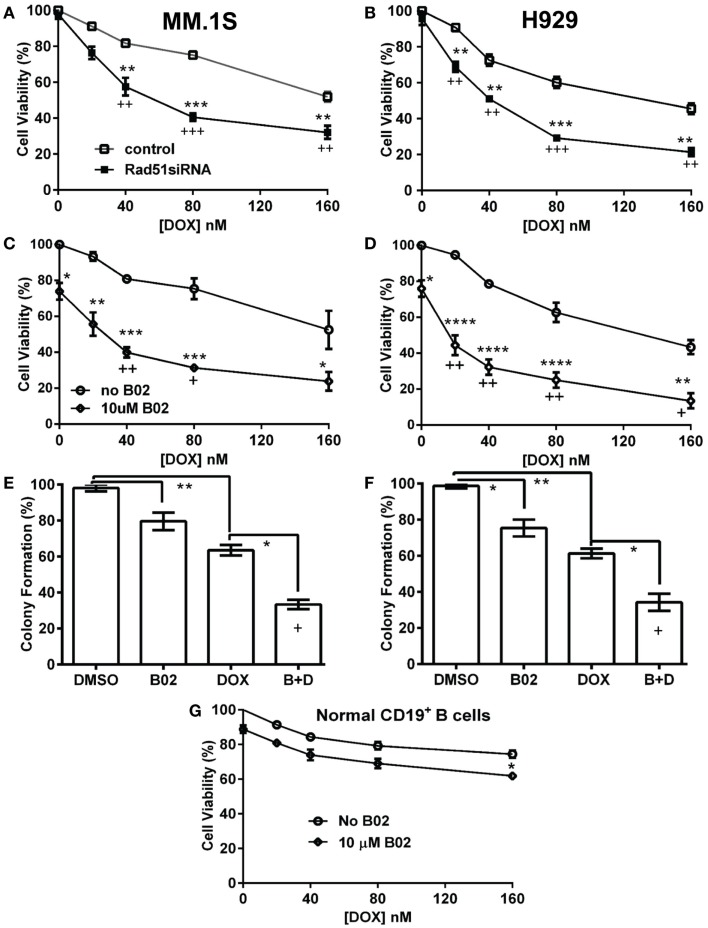
**Rad51 inhibitors potentiate doxorubicin (DOX) toxicity in myeloma cells but not in normal B cells**. **(A,B)** Interaction of DOX with anti-*Rad51* siRNA for toxicity to myeloma cells in mass culture. Cell lines MM.1S **(A)** and H929 **(B)** were treated with DOX at 0–160 nM, 24 h after transfection with plasmid expressing either a *Rad51*-specific siRNA (4) or a scrambled control siRNA. After a further 72 h, cell survival and proliferation were estimated by the WST-1 viable-cell assay (Clontech). **(C,D)** Interaction of DOX with B02, a small-molecule inhibitor of RAD51, for toxicity to myeloma cells in mass culture. Cell lines MM.1S **(C)** and H929 **(D)** were treated with DOX at 0–160 nM, ±B02 at 10 μM; viable-cell number was assessed by WST-1 assay after 72 h. **(E,F)** Effect of DOX ± B02 on colony formation at low density. MM cell lines MM.1S **(E)** and H929 **(F)** were treated with vehicle, DOX (80 nM), B02 (10 μM), or both. Clonogenic survival was assessed in soft agar as described in “Materials and Methods” section. Mean ± SEM is shown for each treatment group, normalized to untreated cells and combined from three independent experiments. **(G)** Viability of normal CD19^+^ B cells from human peripheral blood was assessed by WST-8 assay (Sigma CCK-8). For comparisons of treatment groups connected by brackets, *, **, ***, and **** indicate *p* < 0.05, 0.01, 0.001, and 0.0001, respectively, by heteroscedastic 2-tailed *t*-tests. Significance of synthetic lethality was also tested, by comparing the percentage viability for the combination (B02 + DOX), to the percentage viability predicted by multiplying the surviving fractions after each treatment alone. The coefficient of variation (CoV) for each product of two treatment survivals is the geometric mean of the two individual CoVs. The predicted mean and SD were contrasted to the actual combined-treatment values by a one-tailed heteroscedastic *t* test; **+**, ++, and **+++** indicate synergy *p* values of <0.05, <0.01, and <0.001, respectively.

We first used siRNA to assess whether specific inhibition of Rad51 will enhance DOX chemosensitivity. Anti-*Rad51* siRNA suppressed *Rad51* transcript levels relative to control (scrambled siRNA) by ~65% in MM.1S cells (**A**) and ~61% in H929 (**B**) cells, assessed by qRT-PCR at 24 h (Figure S1 in Supplementary Material). DOX (0–160 nM) was added to cells after 24 h, and cell number was monitored 72 h later as a measure of survival and proliferation. *Rad51* siRNA significantly potentiated DOX toxicity in MM cell lines relative to control siRNA (Figures 1A,B; *p* < 0.01 for seven of eight comparisons), reducing the calculated IC_50_ for DOX by 3.1-fold in H929 cells and by >2.6-fold in MM.1S cells (Table 1).

**Table 1:** **Pre-treatments targeting Rad51 sensitize myeloma cells to DOX**.

Cell line	DOX IC_50_ (nM)		Fold change	DOX IC_50_ (nM)		Fold change
	−B02	+B02		scr-siRNA	*Rad51* siRNA	
MM.1S	>160	25.4	≥6.3	>160	60.5	≥2.6
H929	125	14.9	8.4	126	40.6	3.1
ARP-1	>160	27.3	≥5.9	–	–	–
U266	>160	49.0	≥3.3	–	–	–

IC_50_ values for DOX were calculated from dose-response curves (viability vs. DOX concentration, ±10 μM B02) using GraphPad Prism software. Where DOX did not reach the IC_50_, the lower limit for IC_50_ was taken as the highest dose employed, 160 nM. Fold change is the ratio of IC_50_ values for DOX, “−B02” divided by “+B02” or “scr-siRNA” (scrambled-sequence control) divided by “Rad51 siRNA.”

We then assessed whether B02, a small-molecule inhibitor of RAD51 (31), would enhance myeloma cell sensitivity to DOX. We first determined the cytotoxic dose-response of MM cell lines to B02, to define suitable doses to subsequently combine with DOX. At 10 μM, B02 was moderately toxic (20–24% killing), which was nominally significant for all lines tested (*p* < 0.05, Figures 1C,D and Figure S2 in Supplementary Material) except U266 (*p* > 0.05; Figure S2 in Supplementary Material), which was also the cell line least sensitized to DOX by B02. We then combined 10-μM B02 with a series of DOX doses of increasing toxicity (20–160 nM) to seek enhanced lethality in four MM-cell lines as assessed by cell viability, apoptosis, and clonogenic potential. As shown in Figures 1C,D and Figure S2 in Supplementary Material, this DOX dose range only exceeded 50% toxicity in H929 cells. Combinations of DOX and B02, however, surpassed 50% toxicity in all cell lines, reducing the DOX IC_50_ by 8.4-fold in H929 and at least 6.3-, 5.9-, and 3.9-fold in MM.1S, ARP-1, and U266 cells, respectively (Table 1).

To evaluate whether the above data for MM cells indicate significant synergy (synthetic lethality) between RAD51-inhibition and DOX, we compared cell survival of dual-treatment combinations to the *product* of their individual surviving fractions when given singly, which is the expected effect of combining two drugs that act independently. Because *Rad51* siRNA alone scarcely affected cell-counts in either MM line, relative to cells exposed to control siRNA, the significant decreases in cell survival when this siRNA was combined with DOX are also significantly synergistic, with *p* < 0.01 to *p* < 0.001 (as indicated by “+” symbols in Figures 1A,B). When 10-μM B02 was used to inhibit RAD51, cell number was reduced ~25% in each cell line without DOX addition (Figures 1C,D; Figures S1C,D in Supplementary Material), probably reflecting the dependence of MM cells on RAD51 (for HR repair of routine DNA damage), which is strongly inhibited by B02. DOX toxicity was further elevated by B02 inclusion, achieving significant synergy at 2–4 DOX doses for each MM cell line – i.e., a reduction in cell number beyond that predicted from their individual effects if they were independent (*p* < 0.05 to <0.01, indicated by “+” and “++” symbols in Figures 1C,D; Figure S1 in Supplementary Material).

We next assessed the impact of DOX ± B02 treatments on the viability of normal CD19^+^ B cells from human peripheral blood. As shown in Figure 1G, B02, or DOX alone at the maximum concentrations used did not severely reduce viability of normal CD19^+^ B cells relative to control treatment (89% for 10 μM B02, and 75% for 160 nM DOX). Moreover, DOX failed to reach the IC_50_ either alone or in combination with B02. B02 only slightly enhanced DOX toxicity at its highest dose, producing a nominally significant decrease in viability for both drugs together relative to DOX alone (*p* < 0.05; Figure 1G). These data imply that B02 sensitizes normal myeloid cells to DOX far less than it affects myeloma cells, consistent with increased MM dependence on RAD51.

**Figure 2:**
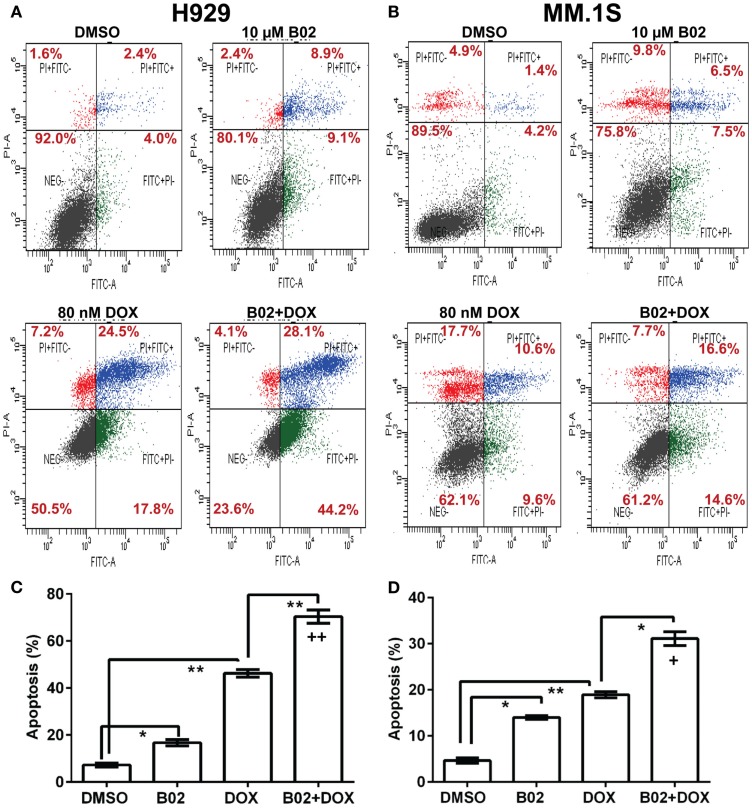
**The RAD51 inhibitor B02 potentiates doxorubicin-induced apoptosis in MM cells**. Myeloma cell lines H929 **(A,C)** and MM.1S (**B,D**) were treated 72 h with vehicle (DMSO), B02 (10 μM), DOX (80 nM), or B02 plus DOX. **(A,B)** The percent of cells undergoing apoptosis was assessed by dual staining with propidium iodide (*y* axis in each panel) and FITC-tagged antibody to Annexin V (*x* axis in each panel). Apoptotic cells are defined by Annexin V content only, and thus are quantitated as the sum of the two right quadrants in each FACS panel. Scatter plots are representative of triplicate samples in each of two independent experiments, comprising 10,000 cells per run scored by flow cytometry. All six replicates of each condition produced similar results. **(C,D)** Combined data are summarized as the mean ± SEM of six data points from two independent experiments (each with *n* = 3). *,**Pairs of treatment *vs*. control groups, connected by brackets, differed significantly (*p < 0.05; **p < 0.01). **+++**, Synthetic lethality was significant (*p* < 0.0003), based on a one-tailed heteroscedastic *t* test comparing the surviving (non-apoptotic) fraction for B02 + DOX combined, vs. the product of the individual surviving fractions after exposure to either agent alone (each corrected for the “background” or uninduced level of apoptosis in cells exposed only to DMSO).

We selected a sub-IC_50_ dose of DOX (80 nM) to test the effect of its combination with 10-μM B02 in assays of colony formation and apoptosis for MM.1S and H929 cell lines. Clonogenic survival of MM.1S cells fell 67% with combined exposure to DOX + B02, rather more than predicted from the 36% decline for DOX and 21% for B02 alone [in terms of viable fractions, 0.33 < (0.64 × 0.79) = 0.51, *p* < 0.03], Figure 1E. Similarly, H929 cells formed 66% fewer colonies with the drug combination, a larger effect than expected from the 39% decrease for DOX alone and 25% for B02 alone [0.34 < (0.61 × 0.75) = 0.46, *p* < 0.04], Figure 1F. Apoptosis assays (Figure 2), like the two measures of cell survival, provide compelling evidence of synergistic killing for the H929 cell line, in which the apoptotic fraction was high, although not in MM.1S with less than half as much apoptosis (Figures 2C,D). In H929 cells, the combination of 10 μM B02 with 80 nM DOX added 66% apoptosis over DMSO vehicle, well above the effect predicted from 12% elicited by B02 alone and 36% by DOX alone [0.34 < (0.88 × 0.64) = 0.56, *p* ≈ 0.0003 for synergy; Figure 2C]. In MM.1S cells, the B02 + DOX combination contributed 26% apoptosis over the level with DMSO alone, significantly greater than 8.4% for B02 or 14.6% for DOX alone (*p* ≤ 0.05, Figure 2D) but roughly equal to the product of their individual effects [0.74 ≈ (0.92 × 0.85) = 0.78, not significant]. With that one exception, all evidence indicates that B02 potentiates the toxicity of DOX for all tested MM cell lines, as indicated by diverse end-points, implying true synergy between these drugs, i.e., a “greater-than-additive” cytotoxic effect.

### Doxorubicin induces increased expression of *Rad51* mRNA and protein, and causes myeloma cells to arrest in S and G2

**Figure 3:**
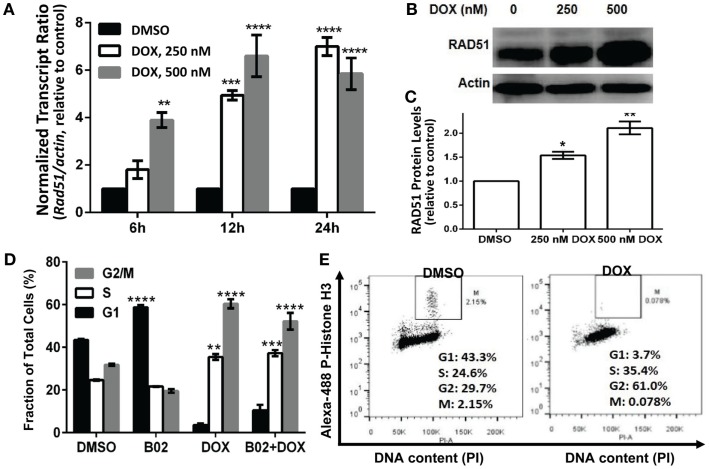
**Doxorubicin effects on MM.1S cells: induction of *Rad51* mRNA and protein, and cell-cycle arrest in S and G2**. Incubation of MM.1S cells with 250 or 500 nM DOX for the indicated periods of time leads to **(A)** increased *Rad51* mRNA levels as assayed by qRT-PCR; **(B,C)** a dose- dependent increase in RAD51 protein level, as shown in western blots; and **(D,E)** cell-cycle arrest, chiefly in G2. **(A)** Data from three independent experiments combined, shown as means ± SEM. Significance of differences between either DOX group vs. DMSO controls (black bars, set to a value of 1), by two-tailed *t* test: *p < 0.05; **p < 0.01; ***p < 0.001; ****p < 0.0001. **(B)** A representative western blot probed with primary antibodies to RAD51 and β-actin. **(C)** Summary of three independent experiments, combined as mean ± SEM (*p < 0.05 or **p < 0.01 relative to vehicle treatment alone). **(D)** Shift in cell-cycle distribution, indicating arrest in S and G2 phases, determined by FACS analysis of relative DNA content per cell (based on fluorescence of DNA with intercalated propidium iodide, in permeabilized cells). Mean values ± SEM are shown for triplicate treatments per experiment for 2–3 biological replicates (total *n* = 6 or 9). Unadjusted significance of differences, relative to the same cell-cycle phase of cells exposed only to DMSO: *p < 0.05; **p < 0.01; ***p < 0.001; ****p < 0.0001. **(E)** Cells treated with DOX or vehicle were stained with propidium iodide and Alexa Fluor 488-conjugated antibody to phosphorylated (ser10) histone H3, to determine the fraction of G2/M-arrested cells that are in mitosis (M).

Homologous recombination occurs predominantly in the S and G2 phases of the cell cycle (37), coinciding with the peak in RAD51 expression (38). RAD51 upregulation can stimulate HR and also may protect against apoptosis (39). In order to assess whether RAD51 itself responds to DOX treatment (or the double-strand DNA breaks it causes), we looked for DOX-induced changes in its transcript- and protein-level expression, or in the cell-cycle distribution, which in turn may alter RAD51 abundance. MM.1S cells were exposed to 250 or 500 nM DOX for 6, 12, or 24 h, before harvesting the cells for analysis. Total RNA was extracted for qRT-PCR quantitation of transcripts, and total protein was prepared for western blotting; the remaining cells were analyzed by fluorescence-activated cell sorting to determine their distribution across the cell cycle. RAD51 expression as mRNA (Figure 3A) and as protein (Figures 3B,C) show consistent induction by DOX treatment, relative to untreated cells. *Rad51* transcripts were dependent on both dose and time of DOX exposure, reaching about sixfold elevation by 12 h at 500 nM, and by 24 h of exposure to 250 nM DOX (Figure 3A). The protein level roughly doubled 24 h post-treatment for the higher DOX dose (Figures 3B,C).

Cell-cycle analysis (Figure 3D) indicates that DOX-treated cells accumulate largely in the S and G2 phases of the cell cycle, whether RAD51 inhibitor was added or not – shifting the total percentage of cells in S and G2 from 54 to 96% for DOX alone and from 57 to 89% for DOX + B02. Since cells in G2 and M phase have the same DNA content and cannot be distinguished by PI staining, we used phosphorylation of histone H3 (on ser10) as a marker for mitotic cells, to partition cells in G2/M into G2 and M phases (40). This revealed that cells exposed to DOX were mainly arrested in the G2 phase with <0.1% in M (Figure 3E), consistent with previous studies showing that DOX prevents human lymphoblasts from traversing G2 (41). In breast cancer and also soft-tissue osteosarcoma cell lines, DOX arrests cells in S and G2, and induces RAD51 expression causing resistance to the drug (19, 20, 42). Pre-treatment with B02 had no significant effect on cell-cycle distribution after DOX exposure, although when added alone it induced a significant increase in G1 arrest (43–59%; *p* < 0.0001) at the expense of G2. We showed previously that RAD51 transcripts and protein are elevated in MM cells compared to normal plasma cells, in the absence of any drug exposure (4). In the current study, we find that RAD51 is further upregulated following chemotherapy with DOX. Based on these observations, in the context of prior evidence that HR repair occurs predominantly in the S and G2 phases (37), and that RAD51 overexpression has anti-apoptotic effects (43), we infer that RAD51 could directly contribute to DOX resistance in MM cells.

### B02 blocks the doxorubicin-induced increase in RAD51 foci, and increases the burden of unrepaired DNA damage

**Figure 4:**
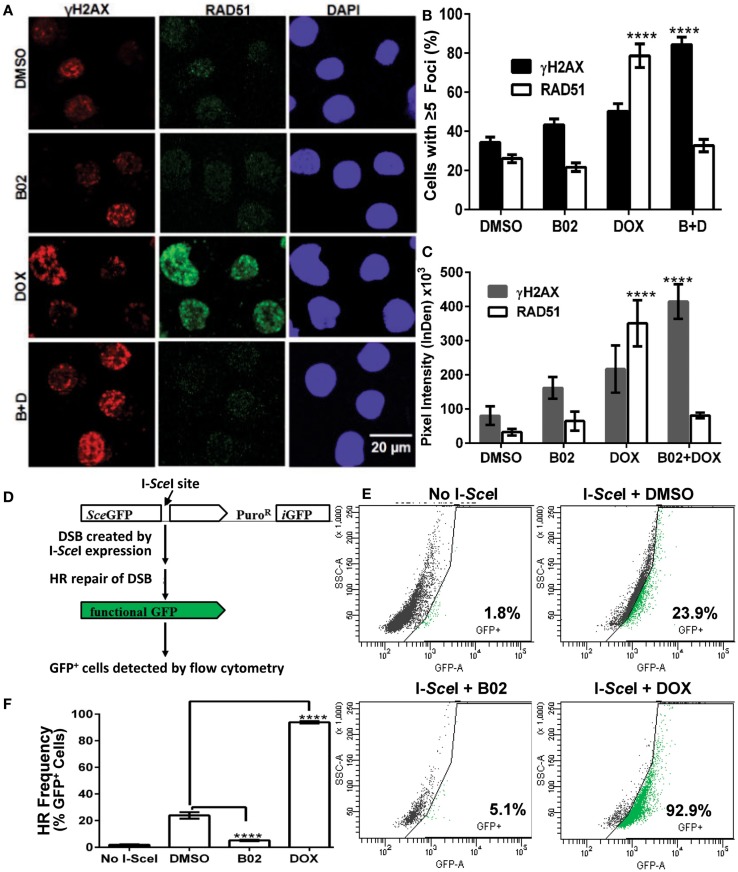
**B02 inhibits DOX-induced formation of RAD51 foci, increases persistence of γH2AX foci, and inhibits HR repair of I-*Sce*I-induced chromosomal DSBs in MM cells**. **(A–C)** MM.1S cells, exposed 24 h to DMSO, B02 (20 μM), DOX (160 nM), or B02 + DOX, were examined by immunofluorescence to identify foci, and DAPI staining to define nuclei. **(A)** Representative images of RAD51 and γH2AX foci in cells exposed to chemicals indicated at left. **(B)** Mean percent of cells with ≥5 RAD51 or γH2AX foci, ±SEM, after the exposures indicated; data were combined from three experiments. ****p < 0.0001 for the effect of each drug treatment, relative to DMSO (vehicle) alone. **(C)** Mean fluorescence (integrated pixel intensity per nucleus) ±SEM, of RAD51 and γH2AX foci after the drug exposures indicated. ****p < 0.0001, as in **(B)**. **(D–F)** B02 inhibits HR repair of I-*Sce*I-induced chromosomal DSBs in MM cells. **(D)** Scheme of HR at a cleaved I-*Sce*I site within the integrated DR-GPF locus. Chromosomal DSBs are first introduced at the single insertion site of the DR-GFP reporter, via cleavage at a unique I-*Sce*I site by site-specific endonuclease introduced by adenovirus infection. HR repair of these DSBs creates intact GFP genes, detected by flow cytometry. **(E)** Examples of flow-cytometric analysis of MM.1S-DR-GFP cells, wherein GFP fluorescence (*x* axis signal) beyond the control boundary (segmented line) indicates HR repair. Background signal (1.8% of cells), defined in cells without I-*Sce*I introduction, rose to ~24% after I-*Sce*I expression. Lower panels show results for I-*Sce*I-exposed cells +B02 (~5% GFP^+^) or +DOX (~93% GFP^+^). **(F)** Summary of combined data from runs such as those illustrated in **(E)**, for cells without I-*Sce*I infection (mock), cells treated with vehicle (DMSO), 20-μM B02, or 160-nM DOX for 24 h after transient infection with I-*Sce*I expression adenovirus (AdNUGS24i). HR data combined from three experiments are presented as means ± SEM. Statistical significance between groups (each *n* = 3) by two-tailed *t*-tests: ****p < 0.0001.

Soon after a DSB is formed, histone H2AX (a variant of H2A) in the region of the break becomes phosphorylated on serine 139. The resulting “γH2AX” sites facilitate recruitment of repair components and chromatin-modulating factors to the DSB vicinity, and consequently nuclear foci that bind antibody to γH2AX are widely used as DSB markers (43). RAD51 foci, in contrast, mark sites where thousands of RAD51 monomers, detectable by immunostaining, have bound single-stranded DNA overhangs at DSBs (32); they thus indicate sites of HR repair for DSBs. The two signals largely colocalize in untreated MM.1S cells with moderate levels of DNA damage (DMSO images, Figure 4A).

Exposure of these cells to DOX (160 nM) tripled the fraction of RAD51 focus-positive nuclei relative to vehicle alone (*p* < 0.0001; Figure 4B), while the total signal per nucleus rose more than 10-fold (*p* < 0.0001; Figure 4C). Exposure of cells to 20 μM B02 had no significant effect on RAD51 foci (Figures 4B,C). However, when many new DSBs were induced by DOX treatment, cells also exposed to B02 could not respond via HR repair, so the number and intensity of RAD51 foci fell well below that of γH2AX foci, to levels not differing significantly from the DMSO control (Figures 4A–C) but much lower than DOX alone (*p* < 0.0001). Low levels of RAD51 foci are associated with a favorable clinical response to chemotherapy (44). γH2AX foci were only slightly increased by DOX treatment alone (*p* > 0.05), whereas the combination of DOX and B02 evoked marked and significant increases in the fraction and intensity of γH2AX-positive nuclei (*p* < 0.0001; Figures 4B,C), indicating a high steady-state level of unrepaired DSBs induced by DOX.

### B02 disrupts HR-mediated repair of DSBs in MM cells

We previously showed high expression of RAD51 and its paralogs, and elevated HR rates, in both MM-cell lines and in primary bone-marrow aspirates from MM patients; the MM.1S cell line in particular overexpressed RAD51 and had consistently robust HR activity (4). We thus chose this cell line to test whether B02 can inhibit formation of RAD51 foci, and thus decrease HR repair. MM.1S cells bearing a chromosomally integrated DR-GFP reporter construct allowed us to measure HR repair in the same cell line (29, 35). This reporter construct contains a *GFP* gene (*sceGFP*) interrupted by an I*-Sce*I cleavage site, and a truncated *GFP* gene (*iGFP*) just downstream of *sceGFP* in the same orientation [Figure 4D and Ref. (35)]. To measure HR, cells were infected with an adenovirus expressing endonuclease I*-Sce*I (AdNGUS24i) (36) to generate site-specific DSBs uniquely within the reporter substrate (since I*-Sce*I sites do not occur elsewhere in the human genome). DSB-repair via HR, using the downstream truncated *iGFP* gene sequence as its template, restores a functional GFP gene – expression of which is detected by flow cytometry.

We assessed HR rates in MM.1S reporter cells treated with B02 or DOX. Cells treated with only vehicle (DMSO) were ~24% GFP^+^, close to the level reported previously for these cells without drug treatment (29). GFP^+^ cells (indicating HR events) increased ~4-fold in DOX-treated cells, relative to vehicle alone (*p* < 0.0001, Figures 4E,F). This may actually represent a >4-fold improvement in HR efficacy, since the fraction of cells repairing the cleaved I*-Sce*I site cannot exceed 100%. In contrast, GFP^+^ cells fell >6-fold after B02 treatment (from 24 to 4%; *p* < 0.0001, Figure 4E). These data (Figure 4) indicate that DOX treatment further increases the already high levels of RAD51 and HR in MM cells, whereas B02 inhibits HR at least sixfold.

## Discussion

Targeting DNA repair proteins has been proposed as a means to selectively sensitize cancer cells to radio- and chemotherapy (45); however, selection of the appropriate target is essential to achieving this goal. We previously reported that RAD51 expression and HR activity are quite generally elevated in MM-cell lines and in primary bone-marrow aspirates from MM patients, and that RAD51 hyperactivity mediates genomic instability and disease progression (4). Others have shown that RAD51 expression in MM patients’ plasma cells correlates inversely with survival (23). NHEJ, the alternative pathway for DSB-repair, appears to be impaired in MM cells (28). These observations imply that MM cells depend on RAD51-dependent HR repair of DSBs, which becomes essential for their survival of DSB-inducing chemotherapies. RAD51 is thus a worthy therapeutic target for inclusion in chemotherapy cocktails to treat myeloma. Although DOX has been widely used for clinical therapy of MM, alone and in combination with other drugs, the ability of drugs targeting HR to boost cytotoxicity at lower doses of DOX has the potential to improve its anti-cancer efficacy while minimizing undesirable side-effects.

In this study, we have demonstrated potentiation of apoptosis, marked reduction of viability, and decreased clonogenic survival of myeloma cells following exposure to relatively low doses of DOX together with a RAD51 inhibitor, B02. While off-target effects of B02 (unrelated to RAD51) are possible, they appear unlikely in view of the similar effect on DOX toxicity to MM cells, of siRNA very specifically targeting *Rad51*. It is especially noteworthy that these synergistic effects of DOX and B02 chemotherapy were substantially greater in MM cells than in peripheral B cells, thus enhancing the therapeutic window for treatment. This preferential toxicity of the DOX + B02 combination for MM cells supports our hypothesis that myeloma cells may be especially dependent on RAD51-mediated HR for survival. In previous studies, agents that indirectly inhibit the expression and/or function of RAD51 were shown to radiosensitize MM cells (30). However, the present study is the first to indicate direct involvement of RAD51 in the chemoresponsiveness of MM cells, in particular to DOX. The clinical use of DOX is limited by dose-dependent cardiotoxicity, which also appears to be mediated by DSBs (46). Combining relatively low doses of DOX with DNA repair inhibitors such as B02 may help mitigate such adverse side-effects.

We showed that DOX treatment induces RAD51 expression and foci, and arrests cells in S and G2, cell-cycle phases wherein HR primarily occurs. RAD51 overexpression and its induction following DOX treatment were previously found to contribute to resistance arising in human soft-tissue sarcoma cells (19). Cell-cycle distribution is an important factor in DOX toxicity, since the drug appears to induce DNA damage (47) and apoptosis (48) chiefly in G2. However, G2 arrest and induction of RAD51 may be protective mechanisms, allowing time for HR repair of the DOX-induced lesions and avoidance of apoptosis (49). Interestingly, we found that addition of B02 to DOX treatment does not significantly alter the extent of G2 arrest or the cell-cycle distribution seen with DOX alone.

We found that DOX induces HR repair, whereas B02 suppresses it without reducing RAD51 foci *unless cells were also treated with DOX* (Figure 4). In the presence of DOX, B02 blocks the DOX-induced increase in RAD51 foci, although the level of unrepaired DSB sites (γH2AX foci) nearly doubles. These data suggest that MM cells have sufficient HR repair capacity to cope with the DSBs induced by 160-nM DOX, but B02 inhibition of RAD51 blocks that repair process and thus exacerbates DNA damage. NHEJ, an alternative DSB-repair pathway, is deficient in myeloma cells; however, NHEJ inhibition in other cell types redirects DSB-repair to HR (28). We have thus confirmed an outcome that was predictable from the above studies: that MM cells should have no effective means of DSB-repair following abrogation of HR (via direct inhibition of RAD51), leading to their more effective killing by drugs that generate DSBs.

Induction of RAD51 after DOX treatment may further increase genomic instability in MM cells due to RAD51-mediated recombination (9) but this effect could be mitigated by co-treatment with RAD51 inhibitors such as B02. RAD51 is essential in proliferating cells, so that its disruption might also be lethal to normal cells. However, *Rad51* is overexpressed in cancer cells relative to normal cells (50, 51) and its selective inhibition by RNA interference increases sensitivity to chemotherapeutic killing of human cancer cells relative to normal cells, both *in vitro* and *in vivo* (24). In the present study, we have demonstrated that a RAD51 small-molecule inhibitor, B02, selectively enhanced DOX killing of MM cells. Thus combination therapies incorporating RAD51 inhibitors along with genotoxic agents such as DOX may offer potential mechanisms to increase chemotherapeutic efficacy.

## Conflict of Interest Statement

The authors declare that the research was conducted in the absence of any commercial or financial relationships that could be construed as a potential conflict of interest.

## Supplementary Material

The Supplementary Material for this article can be found online at http://www.frontiersin.org/Journal/10.3389/fonc.2014.00289/abstract
