# Supplementary figures and images for: A Small-Molecule Inhibitor of RAD51 Reduces Homologous Recombination and Sensitizes Multiple Myeloma Cells to Doxorubicin
Source: Front Oncol. 2014 Oct 30;4:289. doi: 10.3389/fonc.2014.00289 (PMC4214226; doi:10.3389/fonc.2014.00289)

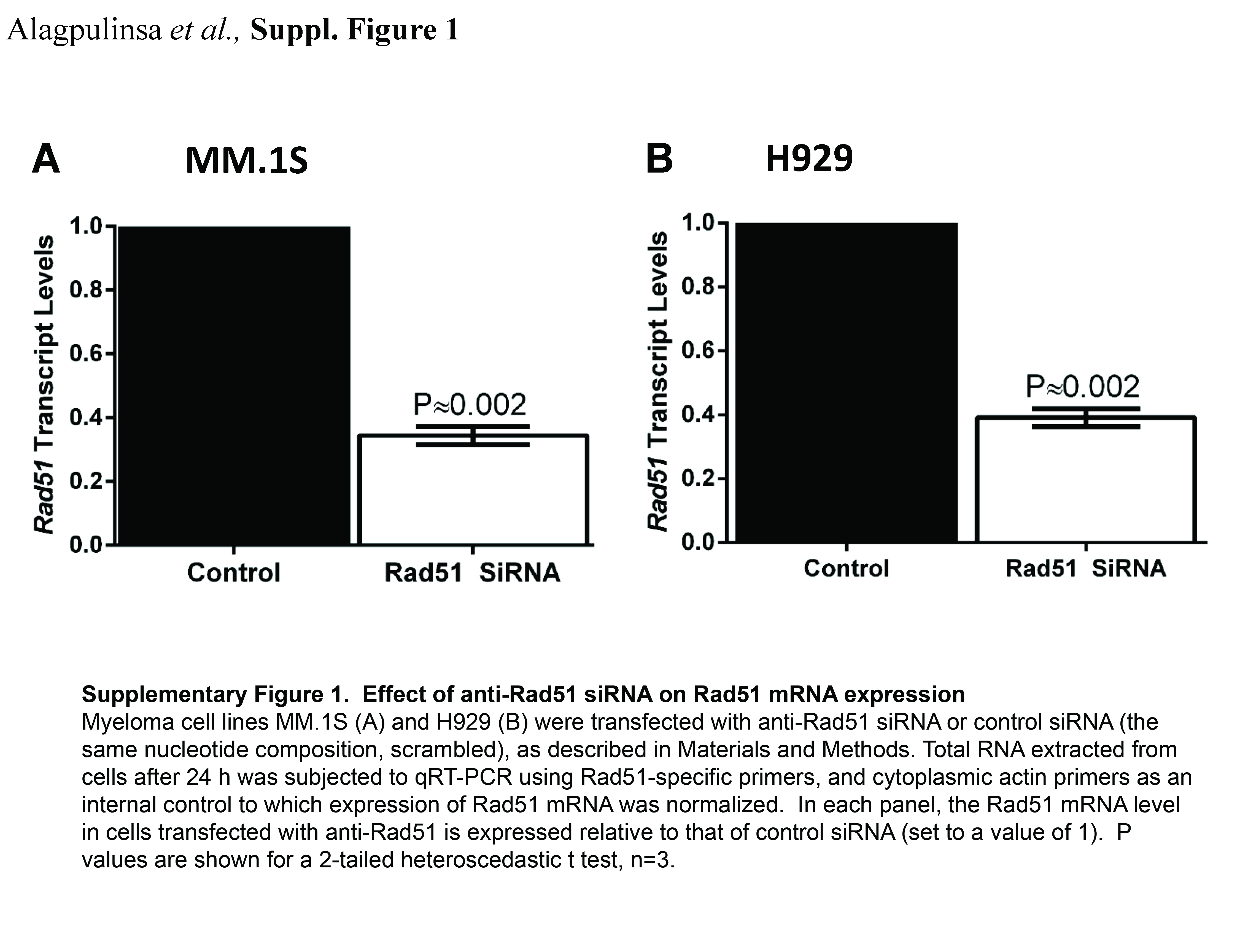

Supplement: Supplementary file 1 [file Image_1.TIF]

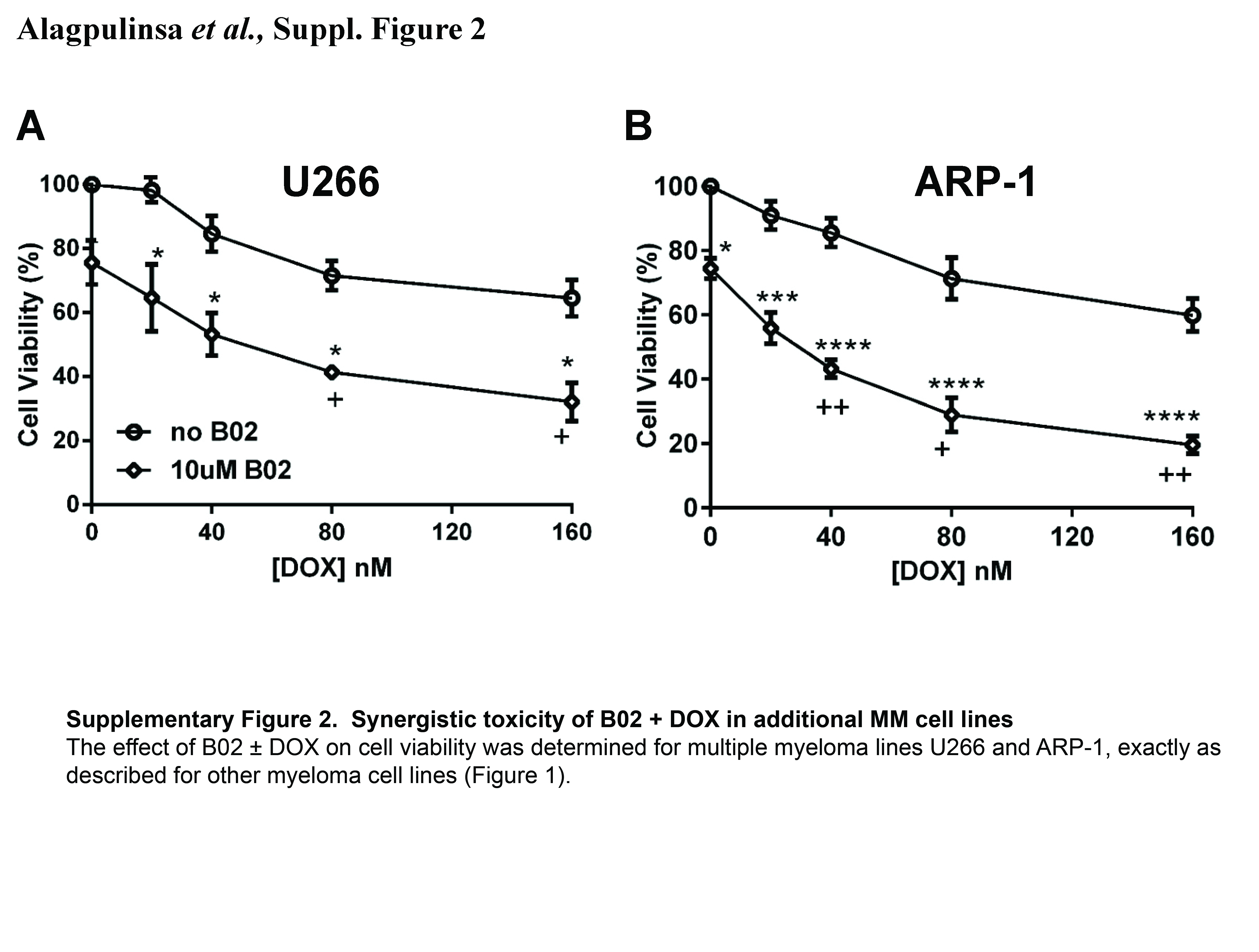

Supplement: Supplementary file 2 [file Image_2.TIF]
